# A case report of giant coronary artery pseudoaneurysm and infective endocarditis: at what level can two pathologies be interconnected on a suitable background?

**DOI:** 10.1093/ehjcr/ytag204

**Published:** 2026-03-13

**Authors:** Gabriela-Adelina Hoinaru, Horea-Laurentiu Onea, Minodora Teodoru, Oana Stoia, Florin-Leontin Lazar

**Affiliations:** Department of Cardiology, County Clinical Emergency Hospital Sibiu, Sibiu 550245, Romania; Department of Cardiology, County Clinical Emergency Hospital Sibiu, Sibiu 550245, Romania; Department of Internal Medicine, Iuliu Hatieganu University of Medicine and Pharmacy, 4th Dept. of Internal Medicine, Medical Clinic No.1, Cluj-Napoca 40006, Romania; Department of Cardiology, County Clinical Emergency Hospital Sibiu, Sibiu 550245, Romania; Medical Clinical Department, Faculty of Medicine, Lucian Blaga University, Sibiu 550024, Romania; Department of Cardiology, County Clinical Emergency Hospital Sibiu, Sibiu 550245, Romania; Medical Clinical Department, Faculty of Medicine, Lucian Blaga University, Sibiu 550024, Romania; Department of Cardiology, County Clinical Emergency Hospital Sibiu, Sibiu 550245, Romania; Department of Internal Medicine, Iuliu Hatieganu University of Medicine and Pharmacy, 4th Dept. of Internal Medicine, Medical Clinic No.1, Cluj-Napoca 40006, Romania

**Keywords:** Coronary pseudoaneurysm, Covered stent, Pulmonary infective endocarditis, Mycotic aneurysm, Case report

## Abstract

**Background:**

Coronary artery pseudoaneurysm (PSA) is a rare but potentially life-threatening condition, typically occurring after percutaneous coronary intervention (PCI). Giant coronary PSA in patients without prior coronary procedures is exceedingly uncommon. Mycotic involvement, particularly with *Staphylococcus aureus*, may accelerate pseudoaneurysm progression and significantly worsen prognosis.

**Case summary:**

We report the case of a 38-year-old man on long-term haemodialysis who presented with unstable angina and new electrocardiographic changes. Coronary angiography revealed severe proximal left anterior descending artery stenosis with a giant PSA. Surgical repair was deemed prohibitive due to marked vascular fragility; thus, emergent PCI with covered stent implantation was performed, achieving satisfactory angiographic exclusion. The patient was discharged in stable condition. One week later, he returned with fever, haemoptysis, and sepsis. Imaging demonstrated a new pulmonary valve vegetation, establishing the diagnosis of infective endocarditis due to *S. aureus*. Despite targeted antibiotic therapy, the vegetation showed progressive enlargement and evidence of embolization. The patient subsequently developed fulminant septic shock and died 12 days after readmission.

**Conclusion:**

This case illustrates the diagnostic and therapeutic challenges of a giant coronary pseudoaneurysm occurring in a haemodialysis patient without prior coronary intervention. Although infection was clinically recognized only after pseudoaneurysm treatment, the later development of *S. aureus* endocarditis raises the possibility that asymptomatic bacteraemia was already present at the initial presentation. The case underscores the importance of maintaining a high index of suspicion for infection in vulnerable patients and of initiating early, individualized management to prevent rapid disease progression and adverse outcomes.

Learning points

- Coronary pseudoaneurysms can develop during asymptomatic bacteraemia, requiring heightened vigilance in high-risk patients, particularly those on long-term haemodialysis.
- Early recognition and treatment of infective endocarditis are essential, as diagnostic delays may result in mycotic coronary pseudoaneurysms and septic embolization.
- Management of coronary pseudoaneurysms should be individualized, and covered stent implantation is a feasible option, but prognosis is poor without rapid infection control.

## Introduction

Coronary artery pseudoaneurysm (PSA) is a very rare but severe condition, most frequently occurring as a post-percutaneous coronary intervention (PCI) complication. The prevalence of giant PSA (defined as more than four times the vessel diameter) in patients without a history of coronary interventions is less than 0.02%. Such cases are most often associated with thoracic trauma or haematogenous infection, including bacteraemia and infective endocarditis, which may occur even in the absence of systemic symptoms.^1^ While in a true aneurysm all three layers of the vessel wall remain intact—although stretched due to structural weakening—PSA involve a defect in the vessel wall, resulting in leakage and a significantly higher risk of rupture.^2^ The differential diagnosis between these two entities can be challenging, and in some cases can only be established after a meticulous integration of clinical, epidemiological, and imaging data.

We report the case of a giant left anterior descending artery (LAD) pseudoaneurysm, likely of multifactorial origin—possibly related to both underlying asymptomatic bacteriemia and local vessel fragility—which was managed percutaneously with a covered stent.

## Summary figure

**Table:** 

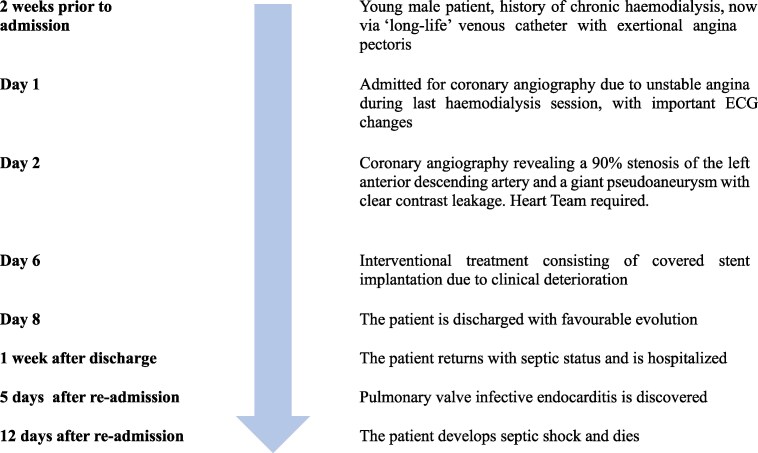

## Case presentation

A 38-year-old male, heavy smoker, with a 22-year history of haemodialysis through a ‘long-life’ venous catheter placed in the right subclavian vein, was admitted to the cardiology department for typical angina associated with new-onset electrocardiogram (ECG) changes. His symptoms had worsened over the previous 2 weeks, and he was scheduled for coronary angiography. Clinical examination revealed an underweight patient with forearm scars from thrombosed arteriovenous fistulas, haemodynamically and respiratory stable, with weak peripheral pulses and anuria.

**Figure 1:**
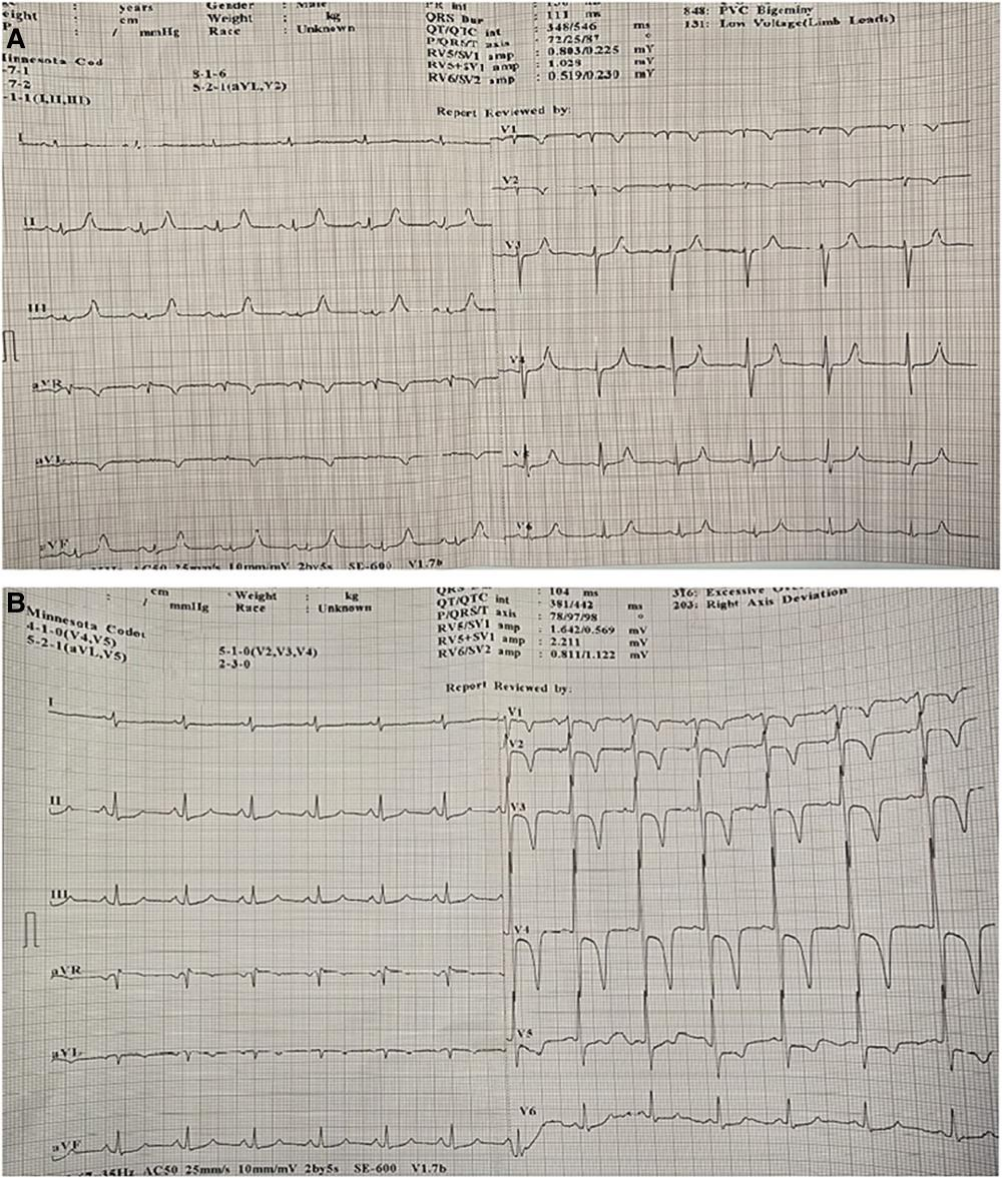
Baseline electrocardiogram pre-intervention, showing sinusal rhythm (*A*) and deep negative T waves in V1-V5 during haemodialysis, associated with chest pain (*B*).

Laboratory tests showed mild anaemia, hypercholesterolaemia, and non-elevated hs-cTnI. The initial ECG demonstrated sinus rhythm with negative T waves in leads aVL and V2. However, during haemodialysis, he developed significant ECG changes, with deep negative T waves in leads V1–V5 (*Figure 1*).

Transthoracic echocardiography (TTE) upon admission showed both ventricles with preserved systolic function, degenerative valvular disease without haemodynamic significance, no pericardial effusion, and no indirect signs of pulmonary hypertension or vegetations. The right atrium was carefully examined for catheter-related thrombi or masses, and no abnormal findings were noted. There was no evidence of interatrial septal defect or shunt by colour Doppler. This baseline evaluation was essential to exclude prior valvular infection or structural heart disease at presentation.

Coronary angiography performed on the third day after admission revealed a 90% stenosis of the proximal LAD, followed by a saccular aneurysmal dilation measuring 23.8 × 23.6 mm supplied through several outlets (see Supplementery material online, *Video S1*). Of note, the patient had never undergone a coronary angiography or PCI in the past, therefore the pseudoaneurysm was interpreted as spontaneous, with no identifiable precipitating cause. The procedure was interrupted and coronary CT angiography was recommended, while the HEART Team was assembled. In the following days, several surgeons considered the operative risk prohibitive (EuroSCORE II = 6.26%, STS Score = 6.4%), mainly due to vascular fragility that had led to complications during multiple prior attempts at femoral vein cannulation for haemodialysis.

**Figure 2:**
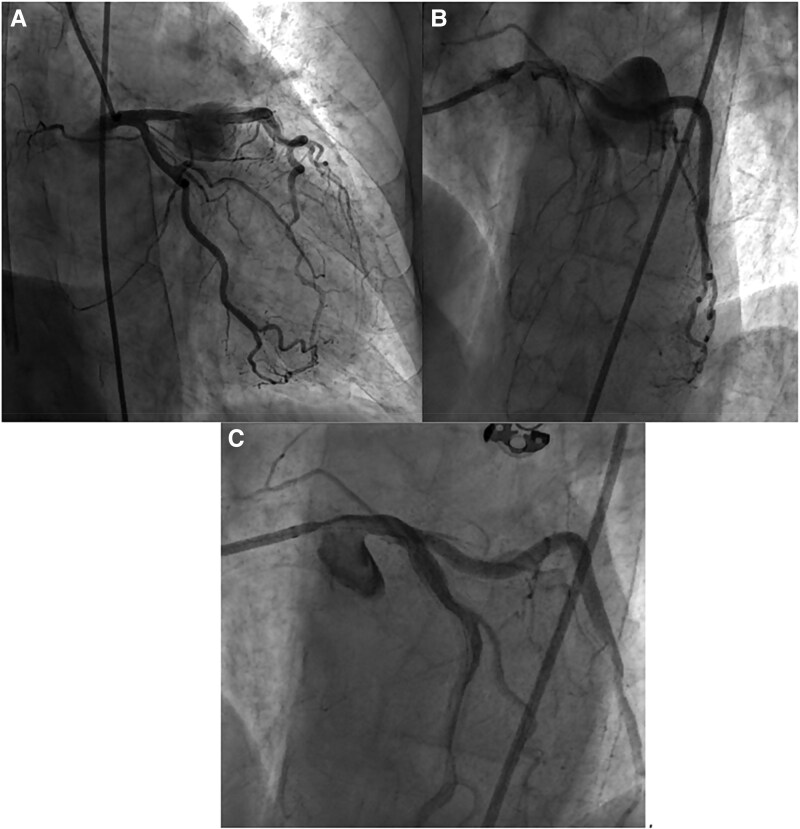
(*A*) Baseline coronary angiography pre-intervention revealed a significant stenosis of the proximal LAD artery and a giant PSA. (*B*) Repeat coronary angiography shows PSA progression. (*C*) TIMI3 flow in the LAD and closure of the PSA after covered stent placement (post-intervention).

Over the next days, the patient experienced increasingly frequent and severe chest pain episodes, leading to the decision to perform emergent PCI of the LAD PSA, despite the coronary CT scan not yet being completed. Repeat coronary angiography 4 days later showed significant enlargement of the PSA (37 × 31.4 mm) (*Figure 2-A,B*, Supplementery material online, *Video S2*). After predilation using a 3.0 × 20 mm semi-compliant balloon, a 3.5 × 30 mm sirolimus drug-coated balloon was applied, followed by implantation of a 4 × 20 mm covered stent from the ostial LAD to just proximal to an important diagonal branch, which was preserved. The rationale for drug-coated balloon use prior to covered stent deployment was to reduce future neointimal proliferation. Notably, intravascular imaging was not available in our catheterization laboratory at that time.

The angiographic result was acceptable (*Figure 2C* and Supplementary material online, *Video S3*), with a small residual outlet in the distal portion of the stent, although the persistence of contrast between injections suggested successful PSA exclusion.

Post-procedural TTE showed no segmental wall motion abnormalities and a preserved left ventricular systolic function. The patient’s subsequent clinical course was favourable; he became asymptomatic and was discharged 5 days after procedure on dual antiplatelet therapy (aspirin 75 mg and clopidogrel 75 mg), with a recommendation for coronary CT angiography.

Approximately 1 week later, the patient presented to the emergency department with impaired general condition, haemoptysis, and fever. Given his 22-year history of haemodialysis through a tunnelled right subclavian venous catheter, the possibility of a catheter-related infection was immediately considered. Although he had shown no signs of infection during the initial hospitalization, patients with end-stage renal disease (ESRD) on chronic catheter-based dialysis are predisposed to *Staphylococcus aureus* bacteraemia and subsequent infective endocarditis, often with a subclinical onset. Therefore, in the presence of new clinical findings, a high index of suspicion for sepsis and infective endocarditis was warranted.

**Figure 3:**
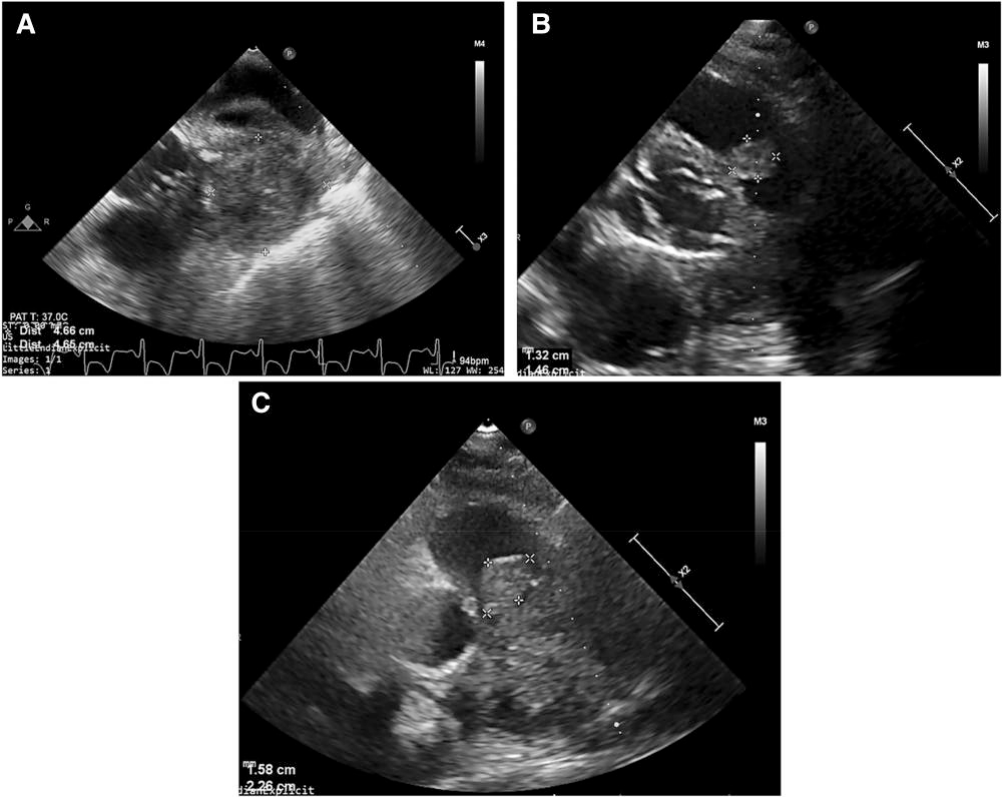
(*A*) Post-intervention transoesophageal echocardiography (modified midoesophageal short-axis view) reveals the partially thrombosed giant PSA (right lateral side of the image), while the mitral valve is free of thrombi or vegetations (left lateral side of the image). (*B*) Parasternal short-axis view reveals a vegetation attached to one of the cusps of the pulmonary valve at re-admission for septic status. (*C*) Parasternal short-axis view at the pulmonary valve level visualizing the vegetation with increasing dimensions after re-admission for septic status.

Laboratory tests showed severe anaemia, marked inflammatory syndrome, and neutrophilia. Both transthoracic and transoesophageal echocardiography (TEE) were performed to evaluate for infective endocarditis. The TEE demonstrating the LAD PSA, partially thrombosed, measuring 47 × 46 mm (*Figure 3-A*), without vegetations on the aortic or mitral valves. The TTE performed at that time did not detect vegetations, thrombi, or abnormal masses on the dialysis catheter, and there was no evidence of interatrial septal defect or shunt.

Empirical antibiotic therapy (gentamicin and oxacillin) was started. Blood cultures later grew *S. aureus*, and therapy was adjusted according to sensitivity testing (tigecycline and meropenem). However, repeat TTE subsequently identified a 13 × 14 mm vegetation attached to one pulmonary valve cusp (see Supplementary material online, *Video S4*), establishing the diagnosis of IE, with two major and two minor Duke criteria fulfilled. Rapid growth of the vegetation was observed despite several antibiotic regimen adjustments (*Figure 3B,C* and Supplementary material online, *Video S5*). Thus, echocardiography was the key diagnostic tool for establishing the final diagnosis of infective endocarditis and for monitoring its progression.

**Figure 4:**
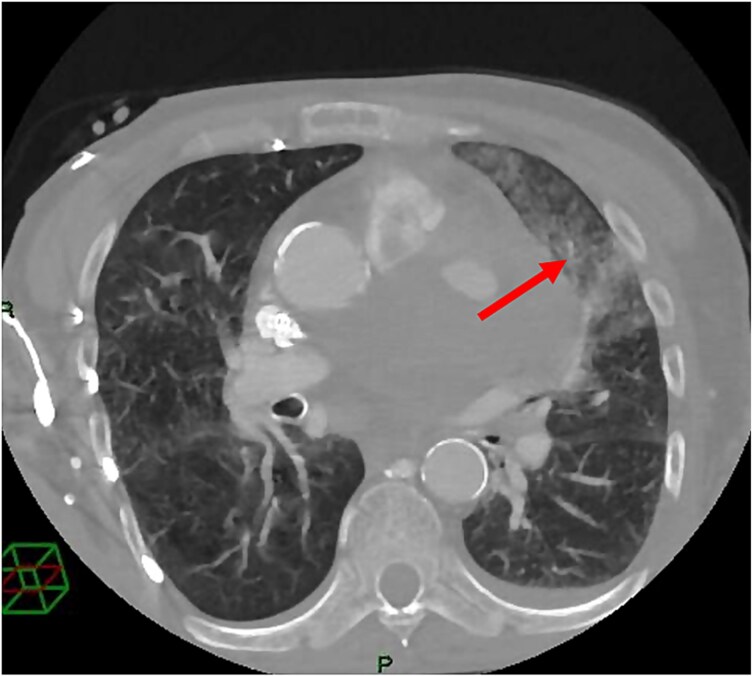
CT scan performed after re-admission for septic status. Area of alveolar haemorrhage (arrow).

**Figure 5:**
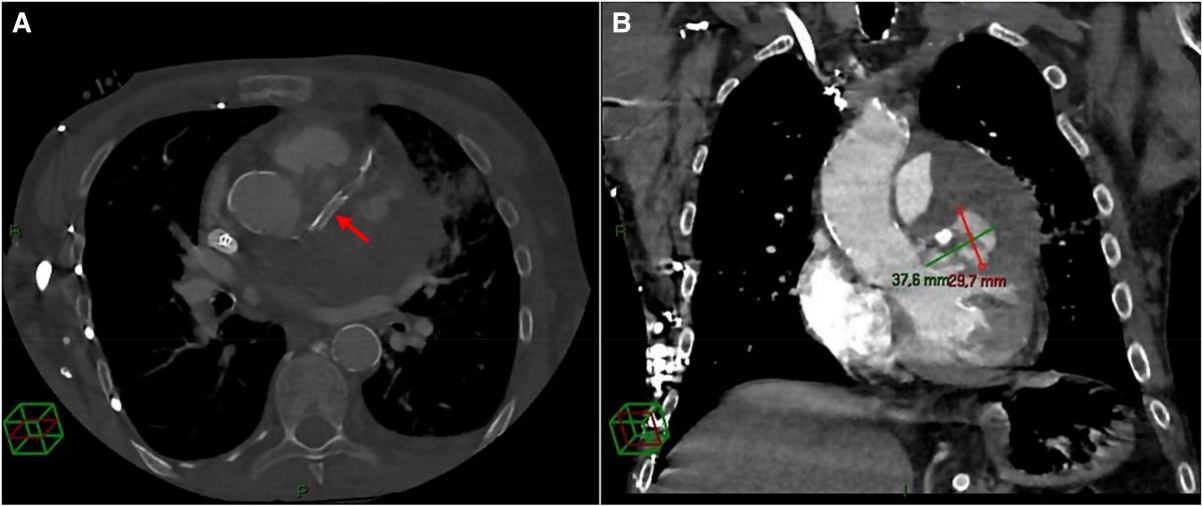
CT scan performed after re-admission for septic status. (*A*) Axial contrast-enhanced CT (bone window) demonstrating a predominantly thrombosed PSA arising from the proximal segment of the stented LAD. The covered stent (arrow) seems well expanded. (*B*) Coronal contrast-enhanced CT (mediastinal window) confirming the 37.8 × 29.7 mm PSA, which contains a substantial mural thrombus surrounding a contrast-filled core, supporting the existence of an active leakage into the PSA.

In the following days, a thoracic CT scan was performed as the patient developed massive haemoptysis. Pulmonary embolism was excluded, and an area of alveolar haemorrhage was identified, interpreted as septic microemboli (*Figure 4*). The LAD stent was visualized crossing a hypodense formation containing areas of contrast uptake, consistent with a PSA with parietal thrombosis and a persistently perfused central cavity (*Figure 5-A,B*).

Replacement of the haemodialysis catheter was considered but postponed due to lack of available alternative vascular access. The patient’s condition rapidly deteriorated: He became haemodynamically unstable, developed septic shock, and ultimately suffered a cardiorespiratory arrest that was unresponsive to resuscitation.

## Discussion

Coronary artery pseudoaneurysms are predominantly caused by atherosclerosis, although Takayasu arteritis, congenital disorders, Kawasaki disease, and PCI have also been described as potential aetiologies.^3^ Multiple histopathological studies have demonstrated hyalinization and lipid deposition leading to disruption of the intimal and medial layers of the vessel wall.^4^ Additionally, mechanical stress and locally increased levels of vasodilatory nitric oxide at sites of stenosis may further weaken the medial wall, contributing to aneurysm formation and, in rare cases, the development of PSA.^5^

Furthermore, mycotic coronary pseudoaneurysms represent another entity that may be associated with IE or even asymptomatic bacteraemia.^6^ The most likely mechanism involves an initial wall stressor—such as atherosclerosis or micro-trauma—followed by bacterial invasion of the vessel wall.^7^ ESRD patients on chronic haemodialysis represent a unique population at especially high risk for infective endocarditis due to repeated vascular access, impaired immune response, and frequent exposure to intravascular devices, with *S. aureus* being the most common etiologic agent.^7,8^ In such patients, even mild or transient bacteraemia should raise suspicion for IE. Comprehensive echocardiographic evaluation—including careful inspection of the dialysis catheter, the right atrium, and the interatrial septum—is therefore mandatory.

The exact mechanism underlying pseudoaneurysm formation in our patient remains uncertain. As the lesion was already present before any percutaneous coronary intervention, it cannot be attributed to procedural trauma. Instead, its development is more likely multifactorial, potentially related to underlying vascular fragility associated with chronic inflammation and possible early or subclinical infection. The subsequent onset of *S. aureus* bacteraemia and pulmonary valve endocarditis further supports the hypothesis of an infectious or haematogenous contribution to pseudoaneurysm progression rather than a purely mechanical cause. Thus, it is reasonable to assume that the asymptomatic bacteraemia contributed to the rapid progression of the pseudoaneurysm, which nearly doubled in size within just 4 days at initial presentation.

Given the limited number of cases described in the literature, no standardized treatment approach exists. Management—whether surgical repair, coil embolization, PCI with covered stent implantation, or conservative therapy—must therefore be individualized. Surgical treatment was discussed by a multidisciplinary Heart Team, but the operative risk was deemed prohibitive due to the patient’s marked vascular fragility, history of rapidly thrombosed arteriovenous fistulas (raising concerns regarding long-term aorto-coronary bypass viability), and a recent haematoma following an attempt at venous catheter insertion. Consequently, the patient underwent PCI with covered stent implantation. Final angiographic assessment revealed a small residual outlet with minimal leakage; however, considering the patient’s young age and the fact that a more distal stent placement would have compromised a major diagonal branch, the result was considered acceptable, and repeat angiography was scheduled for one month later.

The immediate post-procedural course was favourable, and the patient remained symptom-free during hospitalization. He was readmitted 1 week after discharge with sepsis, and blood cultures grew *S. aureus*—the pathogen most frequently implicated in mycotic coronary pseudoaneurysms (approximately 53% of reported cases).^9^ Given the short interval between PCI and the onset of overt infection, it is plausible that subclinical bacteraemia had been present earlier, subsequently leading to endocardial involvement and rapid pseudoaneurysm progression. *S. aureus* is well known for its aggressiveness, and bacteraemia complicated by mycotic pseudoaneurysm and IE carries a particularly high mortality, with delays in either targeted antibiotic therapy or interventional management being associated with poor outcomes.^10,11^

Multimodality imaging plays a pivotal role in both the diagnosis and management of coronary pseudoaneurysms and infective endocarditis in such complex cases. Coronary angiography remains the standard for defining coronary anatomy and planning interventions. CT angiography offers superior assessment of pseudoaneurysm size, wall thickness, thrombosis degree, and relationship with adjacent structures, being particularly valuable for follow-up after covered stent implantation. Echocardiography (TTE/TEE) remains the cornerstone for detecting endocardial infection, identifying vegetations, and monitoring treatment response. In our case, the sequential use of these modalities allowed comprehensive evaluation of both coronary and infective complications, guiding timely interventional and antimicrobial management.

In our patient, the virulence of the microorganism was remarkable, with a fulminant clinical course despite prompt, targeted antibiotic therapy and timely treatment of the coronary pseudoaneurysm. In the context of chronic inflammation and immunosuppression associated with long-term haemodialysis, such an unfavourable evolution may, in retrospect, have been anticipated.

The chronological sequence—initial angiographic detection of the pseudoaneurysm, PCI performed four days later, discharge, and readmission one week afterward with documented *S. aureus* sepsis—suggests that the infection emerged subsequent to, but not as a direct result of, the interventional procedure. The infection became clinically evident only upon readmission, although an asymptomatic bacteraemia may have predated the initial presentation, with the pseudoaneurysm potentially representing an early, unrecognized complication of the infectious process.

In conclusion, this case underscores the need for heightened clinical suspicion and timely evaluation for infective endocarditis in haemodialysis patients presenting with sepsis or new cardiac findings. It also illustrates that coronary pseudoaneurysms and other mycotic complications may arise insidiously during phases of occult or asymptomatic bacteraemia. Early recognition and a multidisciplinary approach remain critical, as even short diagnostic or therapeutic delays can lead to rapid deterioration and poor outcomes.

## Supplementary Material

ytag204_Supplementary_Data

## Data Availability

The data underlying this article will be shared on reasonable request to the corresponding author.

## Lead author biography

Gabriela-Adelina Hoinaru graduated from the Lucian Blaga University of Medicine and Pharmacy, Sibiu, in 2022. She is currently a third-year cardiology resident at the County Clinical Emergency Hospital of Sibiu, with particular interest in multimodality cardiac imaging and advanced heart failure management.

## Notes

**Consent:** The patient has provided written consent for publication, in accordance with the COPE guidelines.
